# Supplementary material for: The Systems Biology Research Tool: evolvable open-source software
Source: BMC Syst Biol. 2008 Jun 29;2:55. doi: 10.1186/1752-0509-2-55 (PMC2446383; doi:10.1186/1752-0509-2-55)
Supplement: Additional file 1 — SBRT Archive. An archive of the current version of the Systems Biology Research Tool. [file 1752-0509-2-55-S1.zip › sbrt-1.4.0/doc/developers_guide/api/sbrt/kernel/KernelProcess.html]

KernelProcess


|  |  |  |
| --- | --- | --- |
| **Overview** \| **Package** \| **Class** \| **Use** \| **Tree** \| **Deprecated** \| **Index** \| **Help** | |  |
| PREV CLASS   **NEXT CLASS** | **FRAMES**    **NO FRAMES**     **All Classes** |  |
| SUMMARY: NESTED \| FIELD \| CONSTR \| METHOD | DETAIL: FIELD \| CONSTR \| METHOD |  |


---


## sbrt.kernel Interface KernelProcess<T>

**Type Parameters:**: `T` - the type of result an instance of this interface produces.

**All Known Subinterfaces:**: MonitoredProcess<R>

**All Known Implementing Classes:**: CatalystDeletionProcess, CdHarFluxVectorProcess, CdHarProcess, CorrelationAnalysis, CvCatalystDeletionProcess, CvFbaOptimizationExperiment, CvFbaOptProcess, CvFluxIntervalProcess, CvObjectiveFunctionProcess, CvRxnDeletionProcess, CycleIdProcess, CycleIdProcess, ExtremeCurrentProcess, FbaOptimizationProcess, FbaProcess, FbaSolverProcess, FluxActivityProcess, FluxCapProcess, FluxIntervalProcess, FluxomeReducerProcess, FluxPlasticityProcess, InitialPointGenerator, IntervalComparison, LinearSolverProcess, MultipleCorrelationAnalysis, MwUAnalysis, NetworkInfoProcess, ObjectiveFunctionAnalysis, PathIdProcess, RandomConstraintsGeneratorV1, RandomEnvGeneratorV1, RandomObjectiveGenerator, RxnDeletionProcess, SingleElementUnionProc, StoichVariationProcess, StrictSingleElementUnionProc, SvCdHarFluxVectorProcess, SvInitialPointProcess, VariableParticipation, VectorComparison, VectorIntersection

---

``` public interface KernelProcess<T> ```

This interface is used to represent processes.
A process is a defined series of actions or operations
used to produce a desired result.

**Author:**
:   This interface was written and documented by
    Jeremiah Wright while in the Wagner lab.

---

| **Method Summary** | |
| --- | --- |
| `void` | `run(ResultsManager<T> resultsManager)`             Runs this process and adds the generated results to the provided results manager via its `ResultsManager.addData(Object)` method. |

**Method Detail**

### run

```
void run(ResultsManager<T> resultsManager)
```

:   Runs this process and adds the generated
    results to the provided results manager via
    its `ResultsManager.addData(Object)`
    method. The method `ResultsManager.breakPoint()`
    may also be called, depending on the type of
    process, but `ResultsManager.close()` will
    *never* be called by this process.

    :   **Parameters:**: `resultsManager` - the results manager to which the results will be added.


---


|  |  |  |
| --- | --- | --- |
| **Overview** \| **Package** \| **Class** \| **Use** \| **Tree** \| **Deprecated** \| **Index** \| **Help** | |  |
| PREV CLASS   **NEXT CLASS** | **FRAMES**    **NO FRAMES**     **All Classes** |  |
| SUMMARY: NESTED \| FIELD \| CONSTR \| METHOD | DETAIL: FIELD \| CONSTR \| METHOD |  |


---
